# Single-Dose Neoadjuvant Pembrolizumab in Resectable Metastatic Melanoma

**DOI:** 10.1245/s10434-025-18314-5

**Published:** 2025-09-12

**Authors:** Neha Shafique, Mohammad Saad Farooq, Valentina Mattfeld, Gracia Maria Vargas, Xiaowei Xu, Lynn Schuchter, Ravi Amaravadi, Tara Mitchell, John T. Miura, Giorgos C. Karakousis

**Affiliations:** 1 Division of Endocrine and Oncologic Surgery, Department of Surgery, University of Pennsylvania, Philadelphia, PA USA; 2 Perelman School of Medicine, University of Pennsylvania, Philadelphia, PA USA; 3 Department of Pathology, University of Pennsylvania, Philadelphia, PA USA; 4 Division of Hematology and Oncology, University of Pennsylvania, Philadelphia, PA USA

**Keywords:** Neoadjuvant immunotherapy, Resectable metastatic melanoma, Melanoma, Metastatic melanoma, Oligometastatic melanoma

## Abstract

**Introduction:**

Neoadjuvant immune checkpoint blockade therapy for resectable oligometastatic melanoma has shown promising results in clinical trials; however, investigation into the optimal agent and dosing schedule is ongoing. We report on the largest case series of patients with oligometastatic melanoma treated with a single dose of neoadjuvant programmed cell death receptor 1 (PD-1) inhibition.

**Methods:**

We identified PD-1 naive patients with resectable stage III/IV melanoma who received one dose of pembrolizumab (200 mg intravenous) prior to surgical resection at a single high-volume melanoma center. Outcomes included time to surgery, 30-day surgical complications, time to initiation of adjuvant therapy, major pathologic response (MPR) defined as < 10% viable tumor, patterns/treatment of first recurrence, and 5-year recurrence-free and overall survival.

**Results:**

Of 51 patients, there were no grade 3/4 immune-related adverse events prior to surgery and no delays in surgery. The majority of patients (70.6%) had no postoperative complications, and none were Clavien-Dindo grade 3 or higher. There was prompt initiation of adjuvant therapy, along with appreciable rates of MPR (19.6%). In total, 45.1% of the cohort experienced a recurrence including two patients who had an MPR. For patients who achieved MPR, 5-year overall survival was 100%.

**Conclusions:**

A single dose of neoadjuvant pembrolizumab is a safe and feasible approach with potential for early pathological readout of responsiveness to neoadjuvant therapy. Late recurrences were observed in the MPR group, indicating need for follow-up but were salvageable. Further biomarker work is needed to identify patients who would benefit from neoadjuvant single agent anti-PD-1 therapy.

Cutaneous melanoma contributes to greater than 65% of all skin cancer-related deaths every year.^1–3^ Importantly, while early-stage melanoma can be successfully treated with surgery alone, more advanced stages are associated with overall worse recurrence and survival rates.^3^ The recent SWOG 1801 and NADINA clinical trials reported superior outcomes using neoadjuvant immunotherapy compared to surgery followed by adjuvant immunotherapy alone for resectable clinical stage III/IV melanoma patients, with practice-changing clinical implications.^4,5^ Notably, these trials used different study designs, treatment agents, and schedules. Neoadjuvant patients in SWOG 1801 received 3 doses of pembrolizumab (anti programmed cell death receptor 1 [PD-1]) prior to surgery with 15 adjuvant doses, whereas in the NADINA trial, patients received two cycles of dual checkpoint blockade with neoadjuvant ipilimumab (anti cytotoxic T-lymphocyte associated protein 4 [CTLA-4]) and nivolumab (anti programmed cell death receptor 1 [PD-1]), with subsequent adjuvant therapy based on pathologic response.

These findings have now been incorporated into clinical guidelines for oligometastatic resectable melanoma.^6^ However, identifying which patients will benefit from neoadjuvant immunotherapy and subsequent choice of agent is vital, particularly owing to the potentially morbid immune-related adverse events (irAE) associated with these drugs and the potential for delay of surgical treatment, which may contribute to worse long-term survival.^7–9^ The optimal agent and dosing schedule for neoadjuvant immunotherapy remains an ongoing area of investigation.

While the SWOG 1801 and NADINA clinical trials have been recent developments, a prior phase Ib clinical trial published by our group evaluated a single dose of neoadjuvant pembrolizumab in addition to 1 year of adjuvant pembrolizumab for patients with resectable stage III/IV cutaneous melanoma in 2019.^10^ A single neoadjuvant dose was sufficient to induce a robust antitumor T-lymphocyte response without any grade 3/4 irAEs and pathologic response was predictive of clinical outcomes.^10,11^ Given the promising efficacy of shortened dosing of anti-PD-1 neoadjuvant therapy, as well as low overall irAE rates, we aimed to further evaluate our expanded cohort with particular attention to postoperative and long-term oncologic outcomes. Real-world validation of these findings and subsequent impact on surgical complications is warranted. In this study, we report on the largest case series of patients with oligometastatic melanoma treated with a single dose of neoadjuvant anti-PD-1 therapy.

## Methods

### Data Source and Inclusion Criteria

This retrospective study was approved by the institutional review board of University of Pennsylvania. We identified PD-1 naïve patients with resectable stage III/IV melanoma who received one dose of pembrolizumab (200 mg intravenous) prior to surgical resection at a single high-volume melanoma center in a prospectively maintained institutional database from 2015 to 2024. Age, sex, race and ethnicity, BRAF status, and surgical procedures were abstracted from clinical records. The type of surgery was categorized as nodal dissection or excision of in-transit, satellite, or subcutaneous metastasis for analysis. Patients that were previously reported in the trial by Sharon et al.^11^ were included in this study, with updated follow-up time and analysis.

### Outcomes and Statistical Analysis

The outcomes studied included time to surgery, irAE prior to surgery, 30-day surgical complications, time to initiation of adjuvant therapy, pathologic response, patterns and treatment of first recurrence, and 5-year recurrence-free survival (RFS), and overall survival (OS).

Time to surgery was calculated from date of neoadjuvant dose to date of surgery. The severity of irAE prior to surgery was graded using Common Terminology Criteria for Adverse Events (CTCAE) on a scale from 1 to 5.^12^ Surgical complications occurring within 30 days were classified according to Clavien–Dindo grade.^13^ Time to initiation of adjuvant therapy was calculated from date of surgery to date of first adjuvant dose. Median follow-up time was calculated from initiation of treatment to last contact and/or death, both without censoring adjustment, as well as using the reverse Kaplan-Meier method accounting for censoring. Pathologic responses to neoadjuvant therapy were classified according to the recommendations of the International Melanoma Consortium (INMC) with major pathologic response (MPR) defined as < 10% viable tumor.^14^ All pathologic assessments were confirmed by melanoma pathologist and co-author Xiaowei Xu during this study. Recurrence and subsequent treatment were recorded when first radiographically or pathologically confirmed from clinical records. Both recurrence-free survival (RFS) and overall survival (OS) were calculated in months from date of surgery.

Descriptive statistics are presented as frequencies for categorical variables and medians with interquartile ranges (IQR) for continuous variables. The Kaplan–Meier method was utilized to calculate RFS and OS and to estimate the effect of pathologic response on survival. All tests were two-sided, and *p* values < 0.05 were considered significant. Analyses were conducted by using Stata version 17 (Stata Corp. LLC, College Station, TX).

## Results

### Demographics of the Cohort

In total, 51 patients met all inclusion criteria (Table 1). The median age was 65 (IQR 57–71) years, and 100% of the patients were White non-Hispanic. The majority of patients (42/51 or 82.4%) underwent a lymph node dissection, whereas the remaining patients underwent an excision of an in-transit, satellite, or subcutaneous metastasis.

**Table 1:** Clinicodemographic characteristics of the cohort

Characteristic	No. patients, *n* (%)
Age, median (IQR), year	65 (57–71)
Sex	
Female	17 (33.3)
Male	34 (66.6)
Race	
White	51 (100)
Ethnicity	
Non-Hispanic	51 (100)
Stage	
III	50 (98.1)
IV (M1a)	1 (1.9)
Type of surgery	
Cervical LND	7 (13.7)
Axillary LND	20 (39.2)
Inguinal LND	14 (27.4)
Parotidectomy	1 (1.9)
WLE of in-transit, satellite, or subcutaneous metastases	9 (17.6)
BRAF status	
Wildtype	21 (41.2)
Mutant	17 (33.3)
Unknown	13 (25.5)

### Perioperative Outcomes and Adjuvant Therapy

There were no grade 3/4 irAE prior to surgery. Two patients experienced irAE prior to surgery with one grade 1 rash and one grade 2 fatigue and liver enzyme elevation. There were no delays in the initially planned surgery date, and 100% of patients underwent surgery with a median time to surgery of 21 days (IQR 14–23).

The majority of patients did not experience any postoperative complications, and none had a complication requiring intervention or intensive care (Table 2). Two patients had a superficial wound dehiscence treated with local wound care. Twelve patients had a superficial surgical site infection requiring oral antibiotics, and one patient had a surgical site infection requiring admission for intravenous antibiotics. There were no seromas that were symptomatic and/or required drainage; however, one patient had a surgical drain placed for > 30 days. All but one patient (98%)—the one who experienced the grade 2 fatigue and liver enzyme elevation prior to surgery—initiated adjuvant therapy with a median time to first adjuvant dose of 23 (IQR 19–29) days.

**Table 2:** 30-day surgical complications

Clavien-Dindo classification	*n* (%)
No complication	36 (70.6)
I (Deviation from ordinary post-operative course without pharmacological or surgical intervention)	2 (3.9)
II (Complication requires pharmacological treatment)	13 (25.5)
III (Complication requires procedural or surgical intervention)	0 (0)
IV (Complications requiring intensive care unit management)	0 (0)
V (Death)	0 (0)

In total, 26 patients (50.9%) did not complete their planned year of adjuvant therapy. The majority of these patients stopped owing to recurrences, but eight patients (16.7%) stopped adjuvant therapy owing to toxicity or intolerance of agent.

### Pathological Response and Survival

**Fig. 1:**
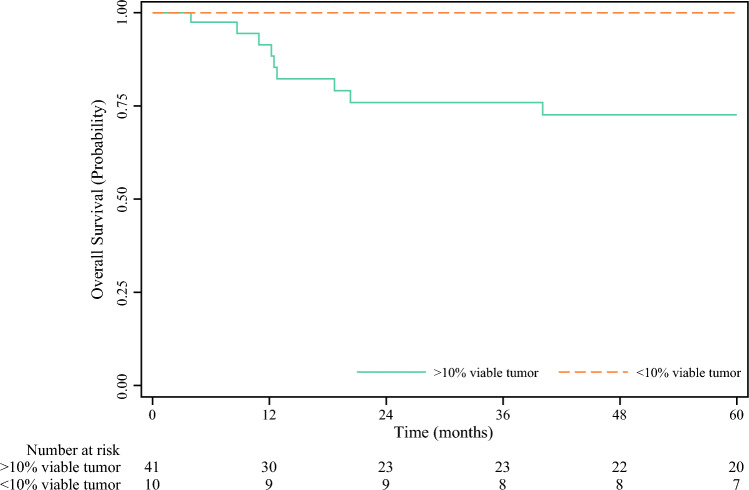
Overall survival stratified by major pathological response (< 10% viable tumor)

In total, ten patients (19.6%) had MPR, and of those, seven patients had confirmed pathologic complete response (pCR). There was no association found between clinical and molecular characteristics known prior to surgery and pathologic response (Table 3). Median follow-up time for the overall cohort without censoring adjustment was 62.5 months (IQR 12–85); additionally, estimated median follow-up time via reverse Kaplan-Meier method was 77 months (IQR 33–92). No deaths occurred in patients who achieved an MPR compared with a 5-year OS of 72.6% for patients with less than MPR (Fig. 1).

**Table 3:** Univariate analysis of patients comparing patients who had a major pathological response (MPR, < 10% viable tumor) compared with those who did not have an MPR

	MPR, *n* (%)	No MPR, *n* (%)	*p*
Age, median (IQR)	63 (50–67)	65 (56–71)	0.79
Sex			
Male	7 (70.0)	27 (65.9)	0.78
Female	3 (30.0)	14 (34.1)	
BRAF status			
Wild-type	3 (30.0)	18 (43.9)	
Mutant	2 (20.0)	15 (36.6)	0.14
Unknown	5 (50.0)	8 (19.5)	
Type of surgery			
LND	9 (90.0)	33 (80.5)	0.12
Excision without LND	1 (10.0)	8 (19.5)

### Timing and Patterns of Recurrences

**Fig. 2:**
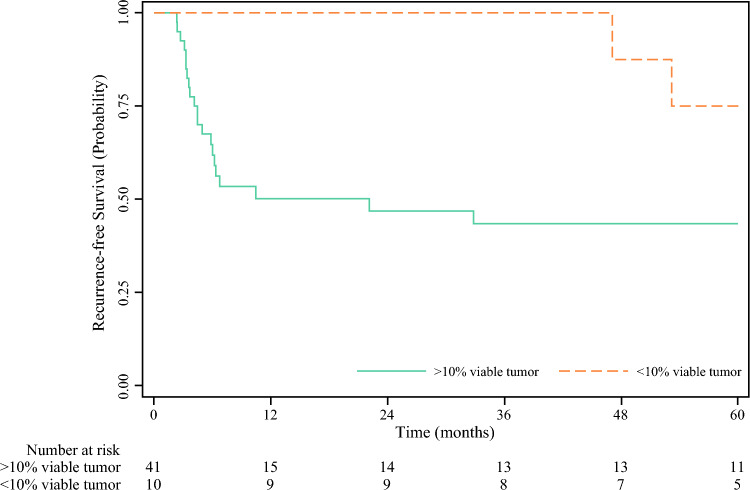
Recurrence-free survival stratified by major pathological response (< 10% viable tumor)

In total, there were 23 recurrences (45.1%) in the cohort; two of these occurred in patients who had an MPR. The recurrences in the MPR group occurred in a delayed fashion with both patients recurring nearly 4 years after surgery (Fig. 2). One patient with MPR and recurrence had early discontinuation of adjuvant pembrolizumab after 2 cycles due to severe rash.

**Fig. 3:**
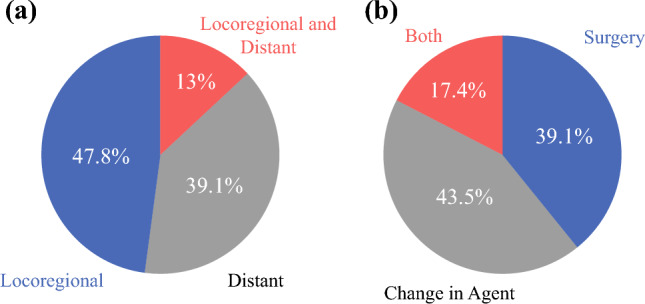
Patterns (**a**) and treatment (**b**) of recurrences

The majority of recurrences were locoregional and were treated in a heterogenous fashion (Fig. 3a). Thirteen patients (13/23; 56.5%) were treated with salvage surgery; four of those patients also underwent a change in therapy, whereas ten patients (10/23; 43.5%) were treated with a new agent or change in agent alone (Fig. 3b). Of note, both recurrences in the MPR group experienced distant metastases (parotid and brain), were treated with local therapy and a new agent, and are alive without disease.

## Discussion

A single dose of neoadjuvant pembrolizumab appears to be a safe and feasible approach with no delays in surgery and prompt initiation of adjuvant therapy, along with appreciable rates of MPR. In the SWOG 1801 trial, after receiving 3 doses of pembrolizumab, 21% of patients had a pCR.^5^ With the three doses, 7% of patients had at least one grade 3/4 irAE prior to surgery and 9% of patients had disease progression or toxicity that precluded surgery altogether.^5^ The results in our study are similar to those demonstrated with the longer neoadjuvant treatment of anti-PD 1 monotherapy demonstrated in SWOG 1801, albeit with the caveat that we specifically report MPR as our primary outcome rather than pCR, and thus an exact equivalence cannot be made. Nevertheless, seven (70%) patients with MPR in our cohort also exhibited confirmed pCR. In summary, these data demonstrate that a shortened neoadjuvant dosing schedule may allow for an earlier pathologic readout of responsiveness to anti-PD 1 monotherapy, while avoiding some of the toxicity seen in both longer dosing schedules and with dual checkpoint blockade.

Importantly, this study represents some of the longest follow-up of a neoadjuvant immunotherapy course to date. A notable finding is that patients who achieve MPR can recur. Surprisingly, the recurrences in both these patients occurred nearly 4 years after surgery; however, both patients were able to be treated and are alive without disease at time of this study. These results demonstrate the importance of long-term follow-up in these patients even in the presence of MPR, as well as the treatment responsiveness of recurrent melanoma. Current National Comprehensive Cancer Network guidelines do not recommend routine imaging to assess for asymptomatic recurrences after 3–5 years^6^; but these data suggest that stopping surveillance imaging at 3 years may miss treatable recurrences.

Given that the patients in this study with MPR had a 5-year OS of 100%, we can reasonably infer that pathologic response can be assessed after a single dose of neoadjuvant immunotherapy and is an important prognostic biomarker for long-term outcomes, even when assessed at an early timepoint. Similarly, a recent study using a single dose of neoadjuvant ipilimumab and nivolumab also resulted in similar pathologic response rates and outcomes to those observed after 2 doses in the NADINA trial.^15^ In this current study, patients continued with adjuvant pembrolizumab for up to a year, regardless of pathologic response.

The improved prognosis after MPR continues to raise the question of whether the extent of surgery or adjuvant treatment can be de-escalated for patients who have MPR. These questions were investigated with the PRADO trial, assessing whether a limited dissection was safe for patients with MPR after neoadjuvant therapy.^16^ Patients with ≤ 10% viable tumor did not undergo further nodal dissection or adjuvant therapy after selective resection of the marked index lymph node. The 2-year RFS for these patients was 93%, suggesting that it may be possible to tailor surgical treatment to pathologic response. Similarly, in the NADINA trial, adjuvant nivolumab was omitted if MPR was achieved.^4^ With dual checkpoint blockade, MPR rates were higher at 59% albeit with significant toxicity (23.1% grade 3/4 irAEs attributed to neoadjuvant treatment). Event-free survival at 12 months was significantly longer in the neoadjuvant group (83.7% vs. 57.2%).

Long-term data for these patients will provide further information regarding the de-escalation of adjuvant treatment in context of MPR. There is also the question of whether and how to appropriately modify treatment for patients who do not achieve an MPR. The NADINA trial allowed for a change to BRAF/MEK targeted adjuvant therapy in patients with BRAF mutant melanoma who were randomized to the neoadjuvant treatment arm but did not have a pathologic response, and also allowed for adjuvant radiation therapy in patients without a pathologic response. These modifications likely further improved outcomes in patients assigned to the neoadjuvant treatment arm of the NADINA study.

Overall, our study supports a role for single-dose neoadjuvant anti-PD-1 for resectable stage III/IV melanoma, as our findings show that it is both safe from a toxicity/surgical standpoint and efficacious. We recommend that the single agent/single dose approach be considered in patients for whom neoadjuvant immunotherapy is indicated, particularly if there is concern regarding potential toxicity from doublet therapy and/or more frequent dosing. Additionally, in patients where there is a concern for presurgical toxicity, or delay in surgery owing to comorbidity/frailty, this approach may be valuable. Notably, to be considered for this approach, patients need a sizeable lesion for confirmatory diagnostic evaluation prior to administration of immunotherapy, which effectively implies a minimum size of 1–2 cm for the tumor. For patients who have bulkier disease and are young/otherwise healthy, a doublet therapy approach may be more reasonable and effective.

There are several limitations to our study. First, this is a retrospective cohort at a single academic institution, which may skew both the complexity of patients that present for treatment and the treatments offered. Additionally, retrospective collection of outcomes/surgical data should be cautiously equated to prospective collection, as in the clinical trials discussed in our study for comparison. Furthermore, while we only included PD-1 naïve patients, both patients with an initial melanoma diagnosis or those with recurrent disease were included, a factor that could potentially have an effect on MPR.

## Conclusions

In the largest case series of patients with oligometastatic melanoma treated with a single dose of neoadjuvant programmed cell death receptor 1 (PD-1) inhibition, there were no delays in surgery and prompt return to adjuvant therapy with appreciable rates of major pathologic response. Pathologic response to neoadjuvant immunotherapy at time of surgery is an important prognostic factor for recurrence-free and overall survival. Long-term follow-up demonstrates that patients with major pathologic responses do develop recurrences, although these recurrences tend to occur later and are salvageable with subsequent treatment, with none resulting in death. Further biomarker development is needed to identify patients who may benefit from neoadjuvant anti-PD-1 monotherapy.
